# Optimization of enhancement-mode MIS-GaN HEMT with dual channel for simple process using TCAD simulation

**DOI:** 10.1038/s41598-026-41105-1

**Published:** 2026-02-25

**Authors:** Kang Hee Lee, Yeonsil Yang, Junseok Heo, Jang Hyun Kim

**Affiliations:** Department of Intelligence Semiconductor Engineering, Ajou University, Suwon, 16499 Republic of Korea

**Keywords:** AlGaN/GaN heterojunction, 2DEG, MIS-HEMT, Dual-channel, IDC-HEMT, ISC-HEMT, E-mode, Body effect, TCAD simulation, Engineering, Materials science, Nanoscience and technology, Physics

## Abstract

A metal-insulator-semiconductor (MIS) GaN high electron mobility transistor (HEMT) utilizing a dual-channel structure is demonstrated for enhancement-mode (E-mode) operation using the Synopsys Sentaurus™ technology computer-aided design (TCAD) simulator. The MIS dual-channel HEMT (IDC-HEMT) employs two AlGaN/GaN heterojunction layers to form two two-dimensional electron gas (2DEG) layers. Electrons in the lower 2DEG layer induce a continuous negative bias body effect on the upper channel, shifting the threshold voltage (*V*_th_) in the positive direction and enabling E-mode operation. This structure achieves E-mode operation without requiring additional complex fabrication steps, such as the precise etching processes used in recessed gate or p-GaN gate designs. The 2DEG sheet density in the upper 2DEG of the MIS single-channel HEMT (ISC-HEMT) and IDC-HEMT are 5.49 × 10^12^ cm^− 2^ and 3.43 × 10^12^ cm^− 2^, respectively, while the lower 2DEG in the IDC-HEMT has sheet density of 0.76 × 10^12^ cm^− 2^, all obtained in the access region with a gate and drain bias of 0 V. Due to the reduced 2DEG sheet density in the upper 2DEG, the proposed IDC-HEMT exhibits degraded performance in on-resistance (*R*_on_), with values 28.7 Ω∙mm, respectively, compared to the ISC-HEMT, which has and a *R*_on_ of 22.7 Ω mm. However, the *V*_th_ of the ISC-HEMT is − 1.41 V, while that of the IDC-HEMT is 0.25 V, demonstrating a significant positive shift of 1.66 V. This confirms that the proposed IDC-HEMT can operate in E-mode.

## Introduction

High electron mobility transistors (HEMTs) have a high two-dimensional electron gas (2DEG) density due to the heterojunction of AlGaN and GaN, which ensures about 1–2 times lower on-resistance (*R*_on_) than Silicon devices. In addition, HEMT shows superior performance in high-frequency, high-power, and high-temperature environments due to their wide bandgap. This makes it suitable for power transistors and power modules using high frequency DC-DC converters^1–4^. HEMTs are classified into depletion mode (D-mode) HEMTs and enhancement mode (E-mode) HEMTs depending on the applied bias. The high conductivity channel naturally generated by the AlGaN/GaN junction causes most HEMT to operate in D-mode. D-mode HEMTs have the disadvantages that the device remains on even at 0 V gate bias. In circuit practice, operating in D-mode raises the risk of fault conditions because a negative gate supply is required, which complicates the power tree and gate-driver design. It also degrades noise immunity, increases leakage current, and thus elevates power consumption^5–7^. By contrast, E-mode HEMTs turn off at 0 V, improving power efficiency and enabling simpler circuit implementations benefits that are particularly attractive for logic applications^8,9^. There are various methods to fabricate E-mode HEMTs, such as p-GaN gate, recessed gate, and fluorinated gate^6,10–15^. The recessed gate structures, which etch the barrier layer of the gate area, destabilize the interface state and affect the stability of the threshold voltage (*V*_th_) and *R*_on_ depending on the etching control. To solve this problem, a method of oxidizing AlGaN at high temperature and etching with KOH has been proposed, but this has the issue of difficulty in mass production due to the slow etching rate^16^. The method of inserting a p-GaN layer under the gate metal has the advantage of not requiring etching the gate area, but the *V*_th_ shift is not sufficient^8^. And the forward junction created between the p-GaN layer and the 2DEG can increase the gate current of the device^17^. Fluorine ion implantation enables a large *V*_th_ shift of the device, but it is difficult to form a uniform ion distribution. There is a problem that the injected fluorine ions can reduce the 2DEG mobility of the GaN layer, resulting in a decrease in the conductivity of the device^8^. The traditional E-mode implementation method presented previously requires an additional process for the device and has limitations in process control. Therefore, sufficient research on dual-channel HEMTs, which feature relatively simple processes, is needed to complement these problems^18,19^.

In this study, we propose an E-mode dual-channel HEMT characterized by a simple fabrication process. Conventional dual-channel HEMTs typically shorten the distance between the source and drain in the lower channel to increase current density without scattering^20,21^. Furthermore, previous studies on E-mode HEMTs with dual channels often involve additional fabrication steps, such as recessed gates or p-GaN gates, to shift the *V*_th_ in the positive direction^22,23^. However, the device proposed in this study does not require such additional processes, making it simpler and more efficient. Unlike conventional dual-channel HEMT designs, the source and drain in the proposed device are positioned exclusively within the upper channel region, utilizing the upper channel as the sole current path while employing the lower channel specifically for *V*_th_ shifting. Additionally, to address potential *V*_th_ instability and significant gate leakage caused by Schottky gate structures, a high-κ dielectric layer is inserted beneath the gate metal. This effectively suppresses gate leakage while ensuring enhanced gate controllability^24,25^. And this study was conducted through the following procedure. To ensure the reliability of the study, an MIS single-channel HEMT (ISC-HEMT) was first fabricated, and TCAD simulations are performed to achieve consistency between the experimental and simulated results for the device structure and current characteristics. Based on this, the device structure is modified to an MIS dual-channel HEMT (IDC-HEMT), and simulations are carried out under the same conditions to quantitatively compare and analyze the characteristics of the two devices.

## Results

### Device structure and fabrication

**Fig. 1:**
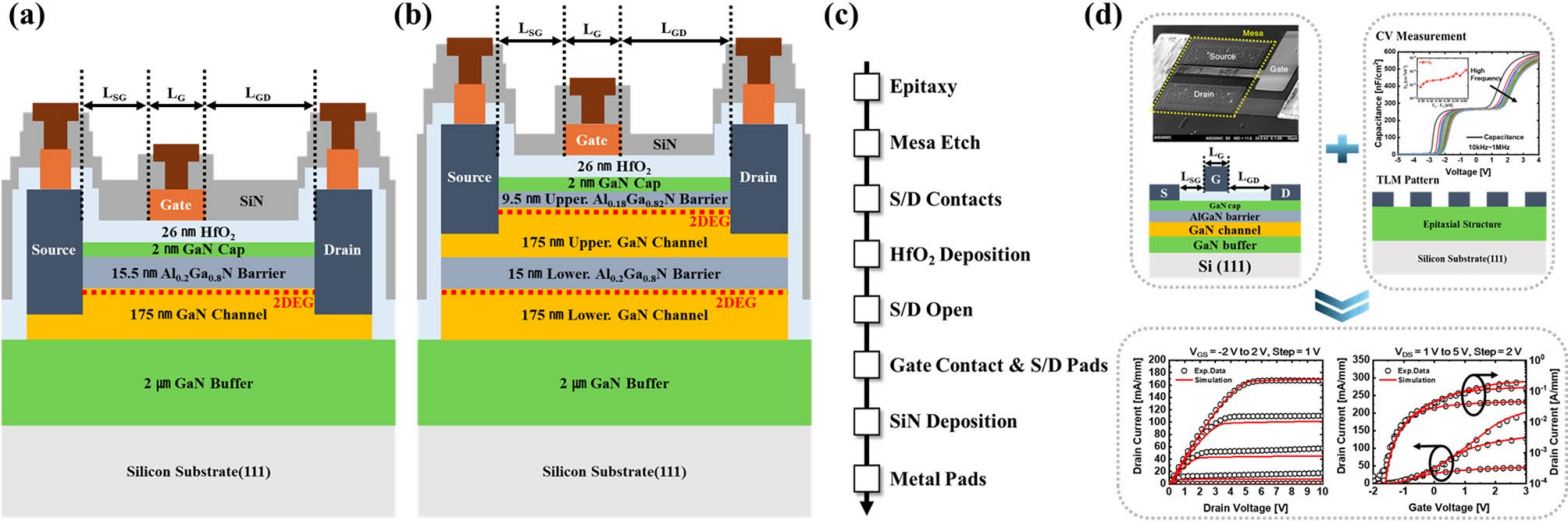
(**a**) Cross-sectional schematic view of ISC-HEMT, (**b**) and IDC-HEMT. (**c**) Device fabrication process flow of the ISC-HEMT and IDC-HEMT. (**d**) Overall calibration process geometric parameters of ISC-HEMT, extracted measurement parameters using CV measurement and TLM, and output curve and transfer curve calibration results of measured and simulated results.

The fabrication process of the ISC-HEMT used in this paper is as follows. The epitaxial structure was processed by metal-organic chemical vapor deposition (MOCVD). Specific epitaxial structure of the device consists of a 2 μm GaN buffer, a 175 nm GaN channel, and a 15.5 nm AlGaN barrier on a silicon substrate, with an Al mole fraction of 20%. Finally, it consists of a 2 nm undoped GaN capping layer. Mesa isolation using inductively coupled plasma reactive ion etching (ICP-RIE) was performed. And the source and drain were composed of Ti/Al/Ni/Au (20/120/55/45 nm) to satisfy the ohmic contact, and then rapid thermal annealing (RTA) was performed at 830 ℃ for 30 s in a N_2_ ambient. The insulator layer, HfO_2_, was deposited 26 nm by atomic layer deposition (ALD) on the entire surface of the device to configure the MIS structure. Then, a photoresist is used as a hard mask to etch the insulator above the source and drain, ensuring that the maintain electrical connection between source-drain metal and contact pad metal. Gate electrode and source-drain metal pads are simultaneously created using lift-off with Ni/Au (20/300 nm). The SiN passivation layer was deposited by plasma enhanced chemical vapor deposition (PECVD). Finally, the gate and source-drain contact pads metal were fabricated as Al/Au (300/50 nm) by lift-off. Additionally, the gate length (*L*_G_) is 10 μm, the distance from gate to source (*L*_SG_) is 5 μm, the distance from gate to drain (*L*_GD_) is 15 μm, and the gate width (*W*_G_) is 200 μm. The fabrication process of the IDC-HEMT is same as the ISC-HEMT except for the epitaxial structure. The epitaxial structure was processed by MOCVD, like ISC-HEMT. The specific epitaxial structure of the device consists of a 2 μm GaN buffer, a 175 nm lower GaN channel, a 15 nm lower AlGaN barrier, a 175 nm upper GaN channel, and a 9.5 nm upper AlGaN barrier on a silicon substrate. Al mole fraction of the lower AlGaN barrier is 20% and Al mole fraction of the upper AlGaN barrier is 18%. Finally, it consists of a 2 nm undoped GaN capping layer. The processes after the formation of the epitaxial structure, such as source and drain formation, gate dielectric deposition, metal pad fabrication, and passivation layer deposition, are identical to those of ISC-HEMT. The specific cross-sectional schematic view and process flow of both devices are shown in Fig. 1a–c. ISC-HEMT and IDC-HEMT differ in forming either a single 2DEG or two 2DEGs, depending on the epitaxial structure. To form two 2DEGs in IDC-HEMT, a GaN channel and an additional AlGaN barrier layer were sequentially grown on top of the conventional ISC-HEMT AlGaN barrier. The RTA process was then employed to create ohmic contacts, allowing the source and drain metal to diffuse and connect to the 2DEG and GaN channel. In IDC-HEMT, the source and drain metal were also connected to the upper GaN channel and 2DEG using the same method. In Fig. 1a and b, the regions of the source and drain metal are depicted to represent electrical connections.

### Simulation details

In this study, ISC-HEMT and IDC-HEMT are simulated by using the Synopsys Sentaurus™ TCAD 2-D device simulator. Various models are employed to analyze electrical characteristics. Since GaN HEMTs have a 2DEG channel, the dominant current component is the drift current determined by the electric field. To account for carrier behavior, the drift-diffusion model and Fermi statistics are used as the carrier transport model, and the self-heating effect is not considered. To address the field changes resulting from variations in the quasi-Fermi levels of electrons and holes due to dopant concentration, the doping-dependence model is adopted as the mobility model, and constant mobility model are used too. Additionally, the high-field saturation model is used to determine electron mobility while considering velocity saturation. To reflect carrier recombination at deep level defect within the bandgap, the Shockley–Read–Hall (SRH) recombination model is applied. The thermionic emission model is implemented for the heterojunction interface, while the bandgap narrowing model is deactivated to ignore the bandgap narrowing effect. To account for polarization effects influencing 2DEG formation, the piezoelectric polarization model is employed. The spontaneous polarization orientation associated with 2DEG formation is set to the Ga-face^26^. The polarization equation used in simulation is given below^27^.

1 $$\:\:{P}_{strain}=2{d}_{31}\cdot\:strain\cdot\:\left({c}_{11}+{c}_{12}-2{c}_{13}^{2}/{c}_{33}\right)$$

2 $$strain=\left(1-relax\right)\cdot\:\left(\frac{{a}_{0}-a}{a}\right)$$

3 $$\:{P}_{total}={\left({P}_{sp}+{P}_{pz}\right)}_{AlGaN}-{\left({P}_{sp}+{P}_{pz}\right)}_{GaN}$$

The d_31_ is piezoelectric coefficients (cm/V) and *c*_11_, *c*_12_, *c*_13_, and *c*_33_ are stiffness constants defined in crystal system (Pa). The *a*_0_ (Å) is strained lattice constant and *a* (Å) is unstrained lattice constant. *relax* is relaxation fitting parameter. All parameters used default values provided by Sentaurus™ TCAD. 2DEG density are calculated by using Eqs. (1), (2) and (3). To enhance the accuracy of the simulations by accounting for quantum effects, the density-gradient model is employed. This consideration is necessary in this study because the AlGaN barrier thickness is below 15 nm. By including quantum effects, the conduction and valence band energy distributions can be regarded as discrete energy levels, allowing a more accurate evaluation of the electron distribution within the channel^27–29^. For source and drain contact robustness, a Schottky work function of 4.3 eV is set. Finally, tunneling mass is set to 0.001 *m*_o_, and electron nonlocal tunneling is activated to mimic ohmic contact behavior^30–35^. The detailed parameters used in the simulation are summarized in Table 1. To enhance the reliability of the simulation results, geometric parameters and measurement parameters obtained from actual experiments were extracted to calibrate the ISC-HEMT. The device was fabricated following the process flow described in previous section, and geometric information, such as the thickness of each epitaxial layer, was extracted. Subsequently for measurement parameters, hall measurement was conducted to extract electron mobility and sheet carrier concentration of the epitaxial structure. Surface donor interface traps were extracted using AC capacitance-voltage (CV) measurements^36^. The results are shown in Fig. 1d. The concentration of the surface donor-like traps was determined to be 2.56 × 10^13^ cm^− 2^, located 0.39 eV below the conduction band energy (*E*_c_). The contact resistivity of the source and drain metals was measured using the transmission line method (TLM), yielding a value of 121.1 mΩ cm^2^. The extracted parameters are fine-tuned for matching the measurement current characteristics. The detailed calibration process flow and calibration results for the output curve and transfer curve are illustrated in Fig. 1d.

**Table 1:** Important geometric, measurement, and model parameters used for calibration.

Symbol	Description	Value
*L*_G_	Gate length	10 μm
*W*_G_	Gate width	200 μm
*L*_GD_	Gate-drain distance	15 μm
*L*_SG_	Gate-Source distance	5 μm
*T*_HfO2_	Thickness of HfO_2_	26 nm
*D*_it_	Surface donor-like trap density	2.56 × 10^13^ cm^− 2^
*E*_it_	Energy level of *D*_it_	E_c_ – 0.39 eV
*ρ*_c_	Contact resistivity	121.2 mΩ cm^2^
*µ*_n_	Electron mobility in GaN	1900 cm^2^/V s
*V*_sat_	GaN saturation velocity	1.8 × 10^7^ cm/s

### Simulation results of ISC-HEMT and IDC-HEMT

#### Comparison of ISC-HEMT and IDC-HEMT

**Fig. 2:**
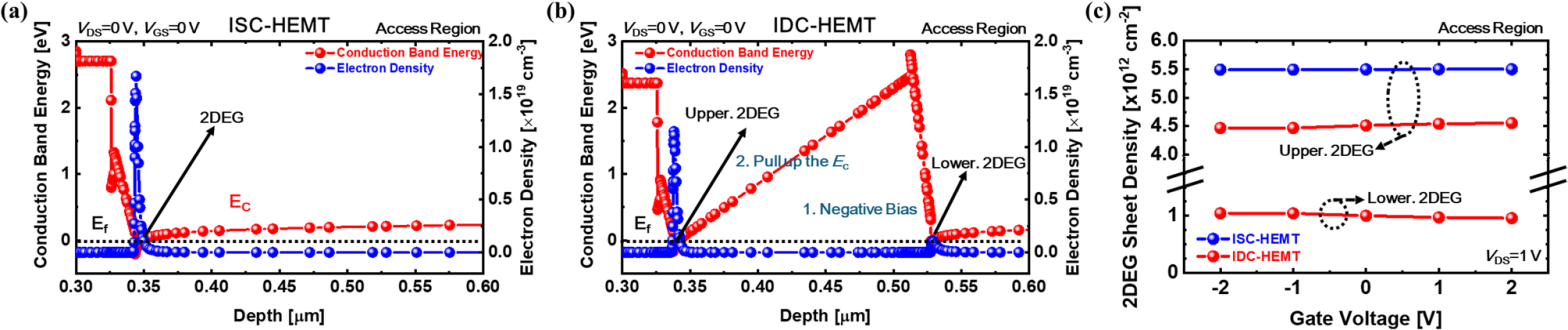
Simulated results of (**a**) conduction band energy diagram and 2DEG sheet density distribution of ISC-HEMT, and (**b**) IDC-HEMT at access region. (**c**) Simulated results of 2DEG sheet change depending on gate voltage conditions. Thickness of AlGaN barrier and GaN channel are identical, 15.5 nm and 175 nm, respectively.

The conduction band energy diagram and electron density distribution at the access region for ISC-HEMT are shown in Fig. 2a, and the results for IDC-HEMT are presented in Fig. 2b. In the IDC-HEMT, two 2DEGs are formed due to the AlGaN/GaN double heterojunction, as indicated by the Fermi level (*E*_f_) rising above the conduction band energy. In contrast, the ISC-HEMT exhibits a single 2DEG formation. The 2DEG electron densities of the upper 2DEG in the IDC-HEMT is 1.15 × 10^19^ cm^− 3^, and the lower 2DEG is 1.10 × 10^18^ cm^− 3^. For the ISC-HEMT, the 2DEG electron density is 1.67 × 10^19^ cm^− 3^. The 2DEG sheet densities of two devices are 3.43 × 10^12^ cm^− 2^, 0.76 × 10^12^ cm^− 2^ and 5.49 × 10^12^ cm^− 2^, following the same order as their corresponding electron density, and all the values are extracted from the access region. To convert the electron density (*n*_s_) into the 2DEG sheet density (*n*_2DEG_), an integration is performed at the position where the potential well is formed. The integration formula is given below.

4 $$\:{n}_{2DEG}={\int\:}_{{y}_{1}}^{{y}_{2}}{n}_{s}\left(y\right)\:dy$$

**Fig. 3:**
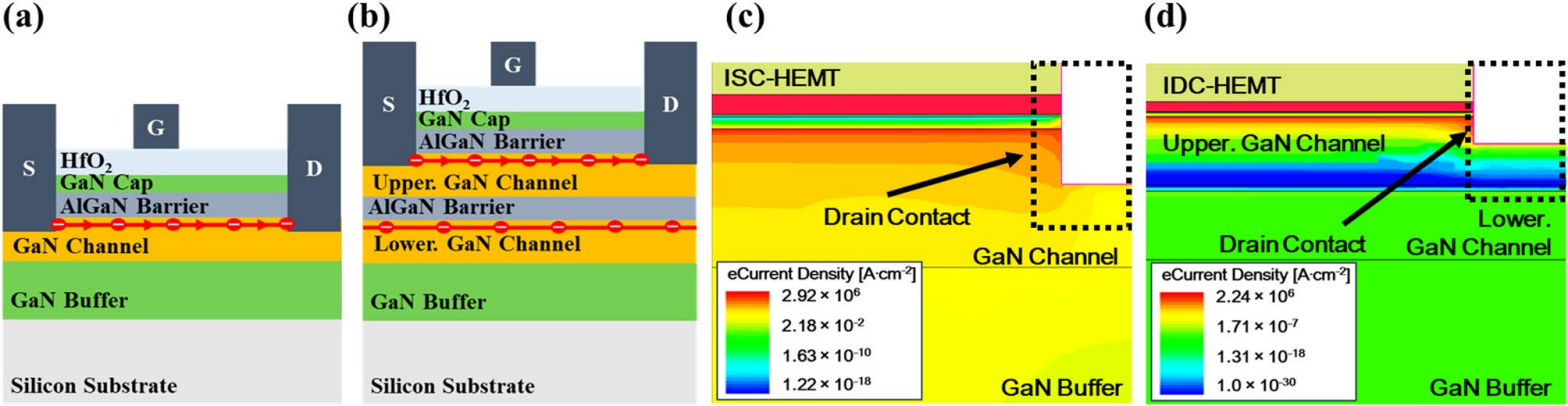
(**a**) Schematic diagram representing the actual operation of ISC-HEMT, and (**b**) IDC-HEMT. Simulated results of (**c**) Electron current density profile of ISC-HEMT, and (**d**) IDC-HEMT.

**Fig. 4:**
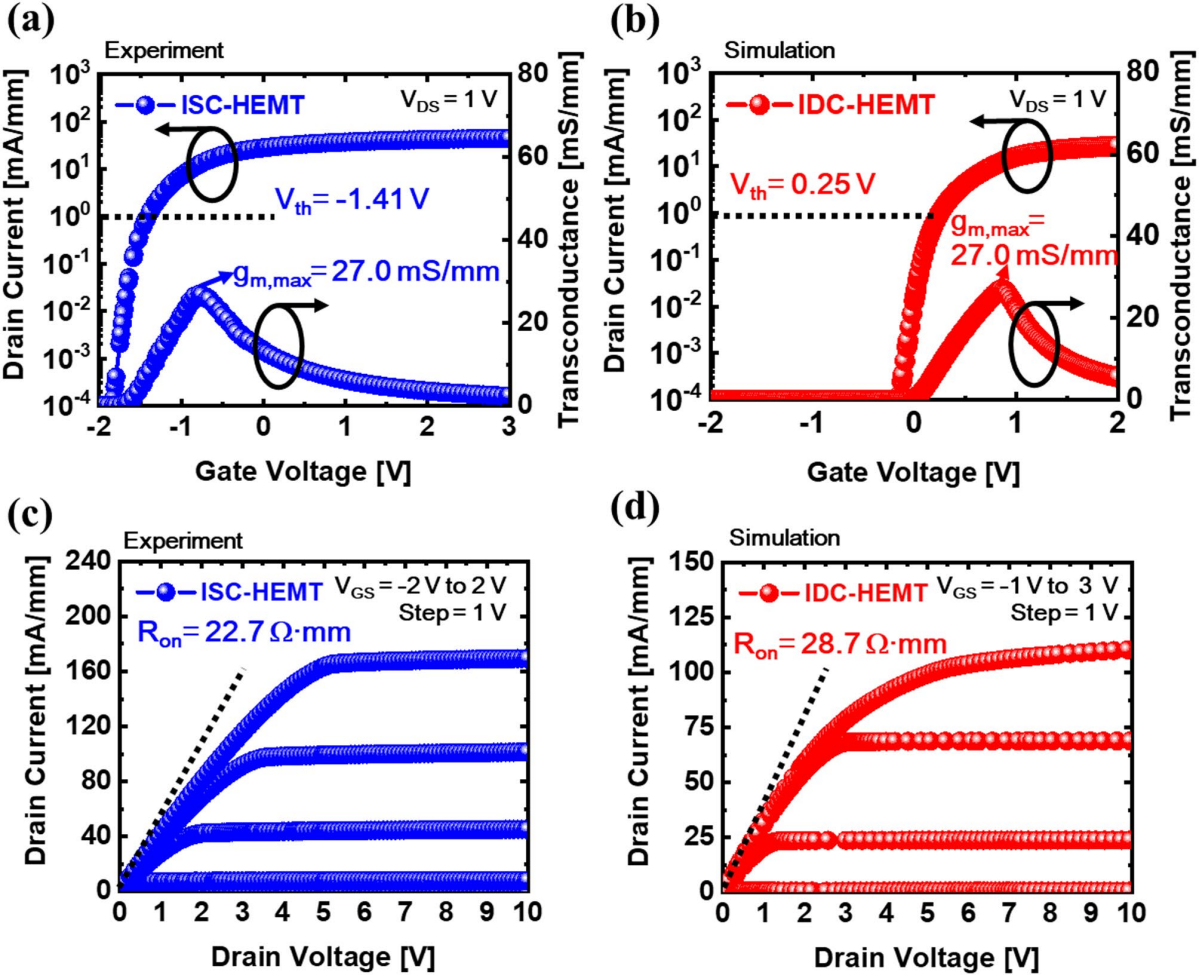
(**a**) Transfer curve and transconductance curve of ISC-HEMT, and (**b**) IDC-HEMT. (**c**) Output curve of ISC-HEMT, and (**d**) IDC-HEMT.

The difference in electron densities of the upper 2DEG between the two devices can be attributed to two reasons. First, variations in the AlGaN barrier thickness and Al mole fraction. A thinner AlGaN barrier weakens the polarization effect, limiting 2DEG confinement and reducing the number of electrons^37–39^. Additionally, the reduction in the Al mole fraction further weakens the polarization effect^40,41^. Second, the effect of the lower 2DEG. Since the 2DEG is fully occupied by electrons, it acts as a region with negative bias. The generated negative bias pulls up the conduction band of the overlying layer. The raised conduction band confines the formation of the upper 2DEG, requiring a larger positive gate bias to eliminate the depletion region in the 2DEG area. Therefore, the lower 2DEG effectively applies a continuous back bias to the upper channel that drives the device. This results in a persistent body effect without requiring additional external voltage. Figure 2b intuitively represents this phenomenon. So, this phenomenon consistently applies across all voltage conditions, shifting *V*_th_ in the positive direction to achieve E-mode operation. Figure 2c shows the impact of the lower 2DEG on the upper 2DEG sheet density in IDC-HEMT as the gate voltage varies from − 2 V to 2 V, compared to the 2DEG sheet density in ISC-HEMT. To ensure a precise comparison, the evaluation is performed in terms of the 2DEG sheet density instead of the electron density. This result demonstrates that the body effect induced by the lower 2DEG persists regardless of changes in gate voltage conditions. To isolate the effect of the lower 2DEG and thin AlGaN barrier, the upper AlGaN barrier and upper GaN channel are set to 15.5 nm and 175 nm, respectively. Schematic diagrams representing the actual operation of the devices are shown in Fig. 3a,b. In the ISC-HEMT, the source and drain are electrically connected to the GaN channel, utilizing the electrons in the 2DEG at that location as the main source for current flow. However, in the IDC-HEMT, the source and drain connection are confined to the upper GaN channel, so that the electrons in the upper 2DEG serve as the main source for current flow. As mentioned earlier, the lower 2DEG in the IDC-HEMT is utilized solely for inducing the continuous body effect. The electron current density profiles for both devices under a gate-source voltage (*V*_GS_) of 5 V and a drain-source voltage (*V*_DS_) of 1 V are shown in Fig. 3c,d. These profiles indicate that current flows through a single 2DEG channel in both ISC-HEMT and IDC-HEMT. Additionally, no current is observed in the lower 2DEG channel of the IDC-HEMT. The transfer curve and transconductance results for ISC-HEMT and IDC-HEMT are shown in Fig. 4a,b. The *V*_GS_ sweep range is from − 2 V to 3 V, and *V*_DS_ is fixed at 1 V. The *V*_th_ of the ISC-HEMT is − 1.41 V, indicating D-mode operation, while the *V*_th_ of the IDC-HEMT is 0.24 V, indicating E-mode operation. The *g*_m, max_ values of ISC-HEMT and IDC-HEMT are 27.0 mS/mm. The output curves are displayed in Fig. 4c,d, with *V*_DS_ swept from 0 V to 10 V and *V*_GS_ ranges determined based on the *V*_th_ of each device. The maximum drain current of the ISC-HEMT is 168.9 mA/mm at *V*_GS_ = 2 V and *V*_DS_ = 10 V while the maximum drain current of the IDC-HEMT is 110.4 mA/mm at *V*_GS_ = 3 V and *V*_DS_ = 10 V. To ensure a precise comparison, the characteristics are compared under the same overdrive (*V*_OV_) condition, showing a resulting ISC-HEMT of 122.9 mA/mm and IDC-HEMT of 110.4 mA/mm at *V*_OV_ = 2.75 V and *V*_DS_ = 10 V. When comparing the *R*_on_ characteristics, the ISC-HEMT has a value of 22.7 Ω mm, while the IDC-HEMT has a higher value of 28.7 Ω mm. This result, similar to the transconductance behavior, indicates slightly poorer performance in the IDC-HEMT. The observed differences in performance between two devices are primarily attributed to the reduction in the thickness of the AlGaN barrier and the lower Al mole fraction in the upper AlGaN barrier of the IDC-HEMT, which reduces the sheet density of the 2DEG in gate and access region. In practice, the DC performance of GaN HEMTs is determined by several factors, including source/drain contact resistance, 2DEG density (*n*_s_), electron velocity (*v*_drift_), and device width (*w*), as can also be seen from the widely used fundamental equations of GaN HEMTs^42^.

5 $$\:{I}_{D}=qw{n}_{s}{v}_{drift}$$

**Fig. 5:**
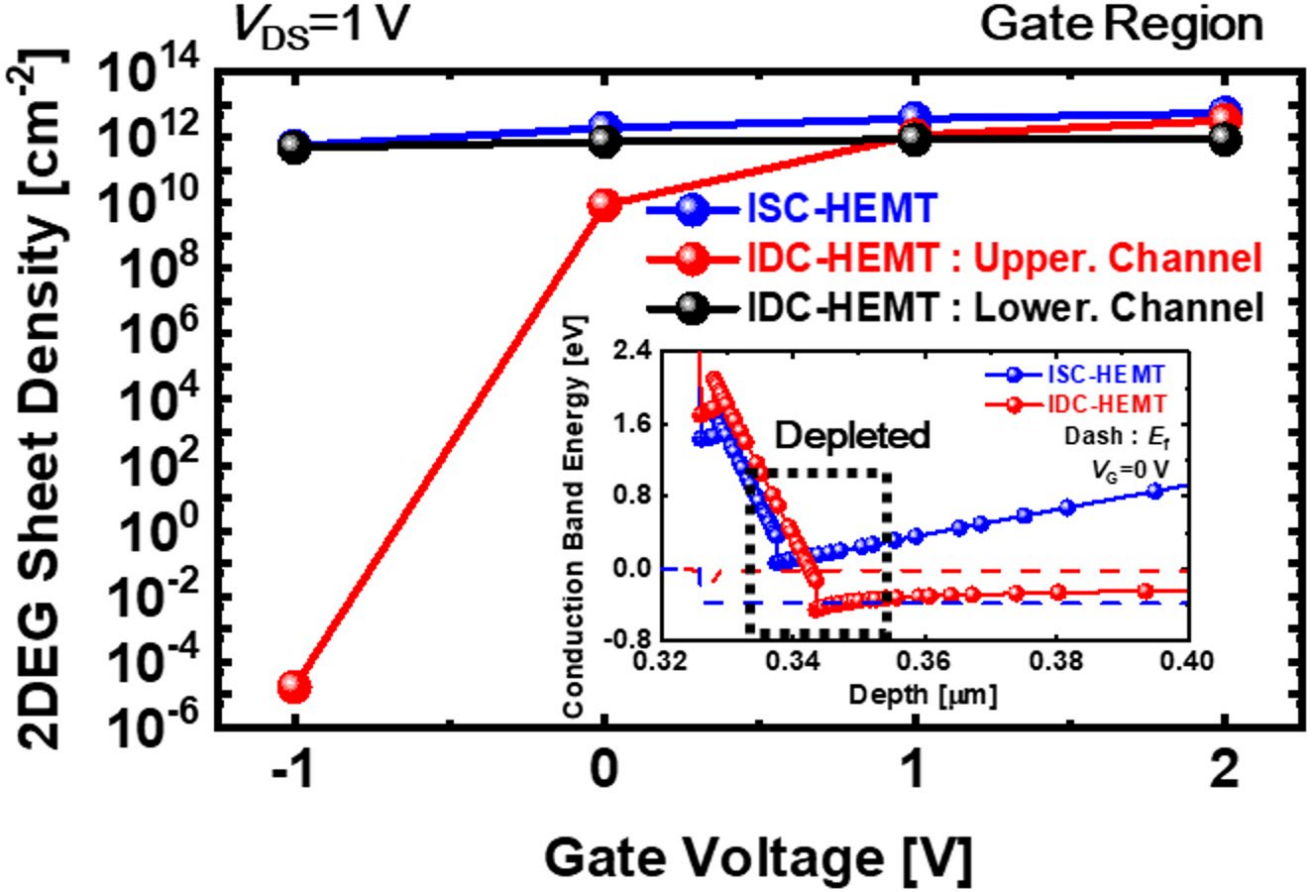
Simulated results of 2DEG density change depending on gate voltage conditions, and conduction band energy diagram at *V*_G_ = 0 V. The structure information of the simulation is shown in Fig. 1a,b, respectively.

**Fig. 6:**
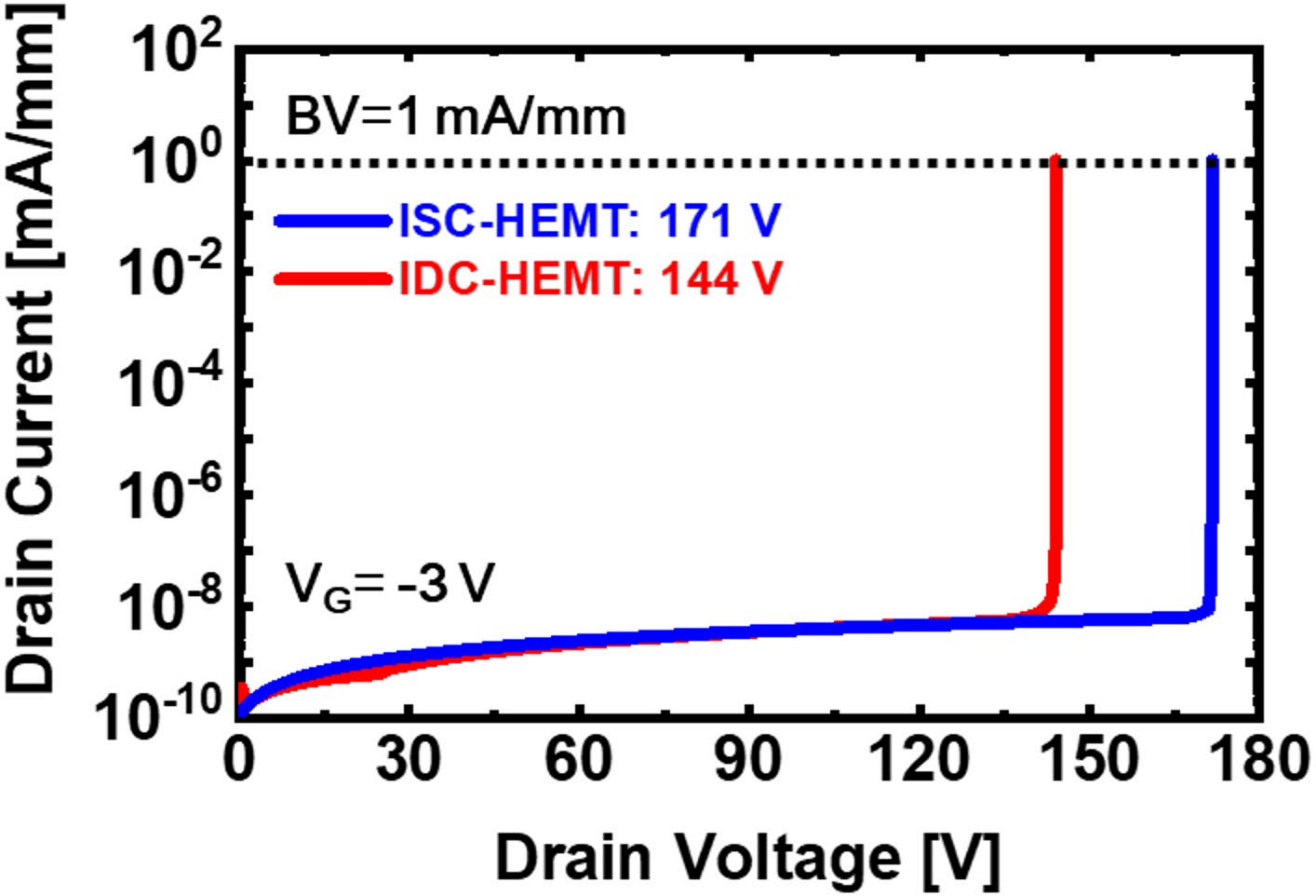
Simulated results of breakdown characteristics of ISC-HEMT, and IDC-HEMT.

In the simulations, IDC-HEMT and ISC-HEMT are configured with the same width and drift velocity. Therefore, the parameter that most significantly affects DC characteristics is *n*_s_. A reduction in the 2DEG density consequently leads to degraded current performance. In this study, in addition to adjusting the lower 2DEG density, the threshold voltage is shifted positively by reducing the AlGaN barrier thickness of the upper channel. During this process, the weakened polarization due to the thinner AlGaN barrier in the upper channel, combined with the body effect of the lower 2DEG, resulted in a simultaneous decrease of the 2DEG density in both the gate and access regions, thereby degrading the current characteristics. Figure 5 shows the 2DEG sheet density as a function of gate voltage and the conduction energy band diagram under a gate voltage of 0 V. In the IDC-HEMT structure, 2DEG sheet density of the lower channel shows negligible variation with gate voltage, indicating operation as a continuous negative bias source for the upper channel without depletion by the gate. The variation in the 2DEG sheet density of the upper channel indicates depletion below 0 V, demonstrating E-mode operation. This is a result of the body effect induced by the lower 2DEG and the presence of a thin AlGaN barrier. The body effect caused by the lower 2DEG can also be observed in the results shown in Fig. 2c. The breakdown characteristics are shown in Fig. 6. The breakdown voltages, defined at 1 mA/mm, are simulated as 171 V for ISC-HEMT and 144 V for IDC-HEMT. The IDC-HEMT exhibited a 22 V lower breakdown voltage due to its double channel structure. Unlike single channel devices, the double channel structure prevents holes generated by impact ionization at the gate-drain edge from escaping into the buffer. Instead, holes accumulate at the upper interface of the lower AlGaN barrier and upper GaN channel. Accumulated holes reduce the source-side potential barrier of the upper GaN channel, allowing electrons from the source to flow more easily. This phenomenon increases the probability of punch-through, leading to a lower breakdown voltage.

#### Optimization results

**Fig. 7:**
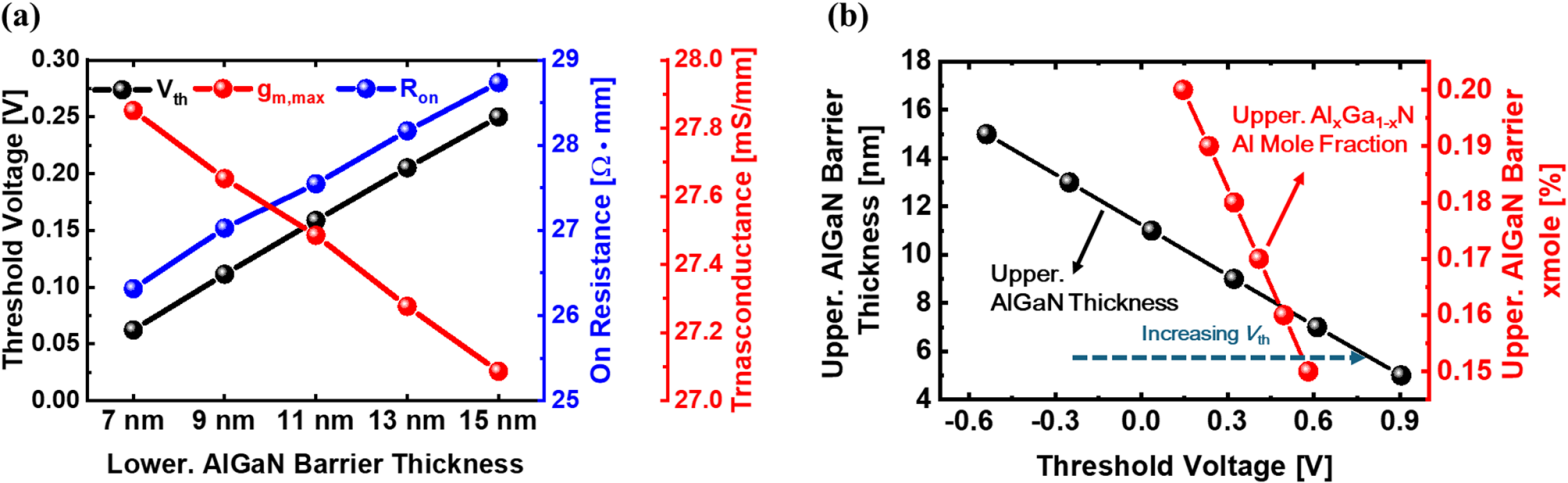
(**a**) Simulated result of *V*_th_, *R*_on_, and *g*_m, max_ distribution according to lower AlGaN barrier thickness of the IDC-HEMT. (**b**) Simulated result of *V*_th_ distribution according to AlGaN barrier thickness and Al mole fraction of the IDC-HEMT.

**Fig. 8:**
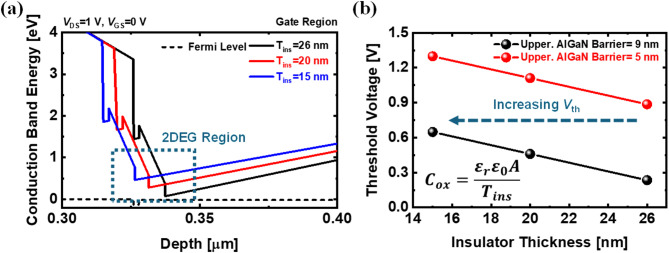
Simulated results (**a**) of conduction band energy diagrams according to insulator thickness and (**b**) *V*_th_ distribution of the IDC-HEMT.

Figure 7 illustrates the effects of the lower and upper AlGaN barrier in IDC-HEMT. The thickness of the lower AlGaN barrier is varied from 7 nm to 15 nm in Fig. 7a. A thicker lower AlGaN barrier enhances the polarization effect, increasing the 2DEG sheet density and strengthening the body effect on the upper 2DEG, thereby shifting *V*_th_ in the positive direction. However, this phenomenon also restricts the formation of the upper 2DEG, deteriorating device performance. Figure 7b illustrates the *V*_th_ distribution of IDC-HEMT as a function of the upper AlGaN barrier thickness and Al mole fraction. The variation range of the upper AlGaN thickness is from 15 nm to 5 nm, with step size of 2 nm. The Al mole fraction variation range is 20% to 15%. As previously mentioned, a thinner AlGaN barrier limits the polarization effect, reducing the 2DEG sheet density. The lower sheet density leads to a smaller current, shifting *V*_th_ in the positive direction. Similarly, a decrease in the Al mole fraction has the same effect, further shifting *V*_th_ positively while deteriorating device performance. The impact of the upper AlGaN barrier is more significant than that of the lower AlGaN barrier. Due to the 2DEG density formed by the upper AlGaN barrier directly serves as the current source. In conclusion, in IDC-HEMT, the characteristics of the AlGaN barrier can be tuned to shift *V*_th_ positively, enabling the optimization of E-mode operation. The *V*_th_ can be shifted positively by enhancing gate controllability through reducing the gate insulator thickness (*T*_ins_). The simulated structure is identical to that shown in Fig. 1b, with the AlGaN barrier set to 9 nm and 5 nm, respectively. As the gate insulator becomes thinner, the gate oxide capacitance (*C*_ox_) increases, leading to more effective depletion at the same gate bias, as shown in Fig. 8a. Consequently, the *V*_th_ shifts positively, and with a 15 nm gate insulator, a stable E-mode operation with a threshold voltage of up to 1.3 V is achieved, as shown in Fig. 8b.

## Discussion

The MIS-HEMT with dual-channel structure, operating in E-mode, demonstrates a unique effect where electrons in the lower 2DEG induce a continuous negative bias on the upper channel. This results in a persistent body effect, enabling E-mode operation. Although the IDC-HEMT shows degraded characteristics in *g*_m, max_ and *R*_on_ compared to the ISC-HEMT, the trade-off is justified by its positive *V*_th_. In circuit design, D-mode devices require the application of a negative bias to the power supply, which increases circuit complexity and the risk of operational failure. Moreover, D-mode devices exhibit higher leakage current, resulting in increased power consumption. Hence, the advantage of E-mode operation is of critical importance when comparing ISC-HEMT and IDC-HEMT. Furthermore, this structure eliminates the need for additional complicated fabrication steps, such as precise etching for recessed gates or p-GaN gate structures, which are commonly employed to achieve E-mode operation. A more detailed comparison of various technologies is presented in Table 2. In summary, the E-mode IDC-HEMT demonstrates clear novelty compared to previously reported dual-channel structures by utilizing only the upper channel as the current path and eliminating the need for additional conventional E-mode processes such as p-GaN layers. This novel approach demonstrates significant potential for future power switching device applications.

**Table 2:** Comparison of various E-mode technologies.

Technology	Strengths	Limitations
p-GaN gate	High *V*_th_, stable E-mode	Process complexity (Etch damage risk), high gate leakage
MIS recessed gate	High *V*_th_, low gate leakage	Process complexity (Depth control, Etch damage risk), unstable *V*_th_
Fluorine implantation	High *V*_th_, process simplicity	Mobility degradation, reliability
IDC-HEMT (this work)	Low gate leakage, process simplicity	*R*_on_ degradation, low breakdown

## Data Availability

The datasets supporting the conclusion of this article are included in the article.
